# Management of abdominal tuberculosis in a community-based hospital in a high-income developing country

**DOI:** 10.1186/s13017-021-00370-3

**Published:** 2021-05-26

**Authors:** Hussam Mousa, Saleh Abdel-Kader, Fikri M. Abu-Zidan

**Affiliations:** 1 Al-Ain Hospital, Al-Ain, UAE; 2 Department of Surgery, College of Medicine and Health Sciences, UAE University, Al-Ain, UAE

**Keywords:** Tuberculosis, Extrapulmonary, Abdominal, Diagnosis, Surgery, Management

## Abstract

**Background:**

The delayed diagnosis and management of abdominal tuberculosis increases its mortality. We aimed to study the clinical presentation, management, and outcome of patients who had abdominal tuberculosis and were treated at Al-Ain Hospital, Al-Ain City, United Arab Emirates.

**Methods:**

All patients who had abdominal tuberculosis and were treated at Al-Ain Hospital between January 2011 and December 2018 were studied. Data were collected retrospectively using a structured protocol including demography, clinical presentation, diagnostic methods, management, and outcome.

**Results:**

Twenty-four patients having a median age of 30 years were studied with an incidence of 0.6/100,000 population. The most common symptoms were abdominal pain (95.8%) and malaise (79.2%). Fever was present only in nine patients (37.5%). Laboratory investigations, except for polymerase chain reaction immunoassay, were not helpful. Chest X-ray was abnormal in three patients (12.5%). Ultrasound and abdominal CT scan were non-specific. Thirteen patients needed surgical intervention for diagnosis or therapy. Diagnosis was confirmed by histopathology in 15 patients (62.5%), immunological assays in 7 patients (29.2%), microbiological culture in 1 patient (4%), and therapeutic trial in 1 patient (4%). The most common type of abdominal tuberculosis was gastrointestinal in 13 patients (54.2%) followed by free wet peritonitis in 5 patients (20.8%). All patients had quadruple anti-tuberculous therapy for a minimum of 6 months. The median hospital stay was 6.5 days. None of our patients died.

**Conclusions:**

Diagnosis of abdominal tuberculosis remains challenging despite advances in medical technology and diagnostic tools. The limited need for diagnostic therapy in our study supports the benefit of PCR assay. Surgery was mainly indicated as the last option to reach the diagnosis or to treat complications.

## Introduction

Tuberculosis is one of the 10 leading causes of death globally, being responsible for the highest mortality caused by a single bacterium. Latent tuberculosis occurs in about one quarter of the world population. In 2019, 10 million people had tuberculosis of whom 1.2 million died [1, 2]. Extrapulmonary tuberculosis accounts for 20% of tuberculosis [3]. Abdominal tuberculosis is the second most common type after pulmonary tuberculosis and accounts for 10–15% of all extrapulmonary cases [4, 5].

It is a misconception that abdominal tuberculosis is a disease of poor communities because it may occur in developed countries, especially in immunocompromised patients [5]. This disease can be transmitted to the gastrointestinal tract by swallowing the infected sputum to the lymph nodes and solid organs by lymphatic and hematogenous spread; and to the internal organs, such as the fallopian tubes and adnexa in females, from adjacent infected foci [6–8]. Abdominal tuberculosis is a great mimicker for many diseases, such as malignancy and Crohn’s disease, and this may present a major challenge for diagnosis if it is not suspected [7–9]. Delayed diagnosis and treatment increase mortality by about 10% [10]. We aimed to study the clinical presentation, diagnosis, management, and outcome of patients who had abdominal tuberculosis and were treated at Al-Ain Hospital, Al-Ain City, United Arab Emirates.

## Patients and methods

### Ethics approval and consent to participate

Ethical approval was given by Al-Ain Hospital Research Ethics Committee, Al-Ain, UAE, (Ethical approval number: AAHEC-04-20-009). Patients’ informed consent was not required as the study was retrospective. Personal patient identifiers were protected during data collection.

#### Study setting and population

Al-Ain Hospital was the main acute care surgery center in Al-Ain City, having 550 beds until March 2021 when it was designated the COVID-19 care hospital of the city during the COVID-19 pandemic. The population of Al-Ain City was estimated to be around half a million during the study period [11].

#### Inclusion criteria

All patients who had abdominal tuberculosis and were treated at Al-Ain Hospital between January 2011 and December 2018 were included.

#### Data collection

Data were collected retrospectively. A special protocol was developed, tested, and refined before collecting the data. Data were collected by an experienced surgeon (S A-K) who was instructed and trained by the senior author (FAZ) to assure the accuracy of data collection. Collected data included demographics (age, gender, nationality), clinical presentation, diagnostic methods, type of abdominal tuberculosis, management, and clinical outcome.

#### Statistical analysis

Data were presented using summary descriptive statistics including median (range) for continuous or ordinal data and number (%) for categorical data. Missing data were not imputed, and percentages were calculated from available data. Incidence was calculated by dividing the annual number of cases by the city population during the study period and was expressed as cases per 100,000 inhabitants. We used the Statistical Package for the Social Sciences (IBM-SPSS version 26, Chicago, IL) for statistical analysis.

## Results

### History and clinical findings

Twenty-four patients having a median (range) age of 30 (19–59) years were studied. Thirteen (54.3%) were females (Table 1). The incidence of abdominal tuberculosis was 0.6 per 100,000 population. The most common presenting symptoms were abdominal pain (95.8%), malaise (79.2%), and loss of appetite (79.2%). Fever was present in only nine patients (37.5%). The median duration of symptoms prior to seeking medical care was 2 months. Five patients had a comorbidity or previous disease (20.8%): one had previous lung tuberculosis, one had lung tuberculosis and diabetes, one had lung tuberculosis and colonic adenocarcinoma, one had a desmoid tumor of abdominal wall, and one was hypertensive with chronic renal disease. HIV test was done in 14 patients and was negative in all. The most common clinical findings were abdominal tenderness (75%) and distension (37.5%) (Table 2). Only two patients presented with a clear picture of peritonitis (8.3%). The most common type of abdominal tuberculosis was gastrointestinal (54.2%) followed by free wet peritonitis (ascites) (20.8%) (Table 3). Abdominal tuberculosis was suspected in only 13 patients (54.2%) on admission (Table 4).

**Table 1:** Demography of 24 patients with abdominal tuberculosis treated at Al-Ain Hospital during the period January 2011 to December 2018

Variable	Value
**Gender**	
Female	13 (54.2%)
Male	11 (45.8%)
**Age**	30 (19–59)
**Nationality**	
Philippines	6 (25%)
Ethiopia	5 (20.8%)
India	5 (20.8%)
Bangladesh	3 (12.5%)
Oman	2 (8.3%)
Other	3 (12.5%)

Data are presented as median (range) or number (%) as appropriate

**Table 2:** Symptoms and signs of 24 patients with abdominal tuberculosis treated at Al-Ain Hospital during the period January 2011 to December 2018

Variable	Number (%)
**Symptoms**	
Abdominal pain	23 (95.8)
Malaise	19 (79.2)
Loss of appetite	19 (79.2)
Nausea	16 (66.7)
Loss of weight	15 (62.5)
Diarrhea	13 (54.2)
Fever	9 (37.5)
Night sweats	6 (25)
Vomiting	5 (20.8%)
**Signs**	
Tenderness	18 (75)
Abdominal distension	9 (37.5)
Guarding	8 (33.3)
Ascites	8 (33.3)
Rigidity	2 (8.3)
Negative bowel sounds	2 (8.3)

**Table 3:** Type of abdominal tuberculosis in 24 patients treated at Al-Ain Hospital during the period January 2011 to December 2018 and their diagnostic workup

Workup	Gastrointestinal *N* = 13	Wet peritonitis *N* = 5	Dry peritonitis *N* = 4	Lymphadenopathy *N* = 2	Total *n* = 24 Number (%)
Immunoassay	9	3	1	1	14 (58.3%)
Ultrasound	7	4	2	1	14 (58.3%)
CT Scan	7	5	2	2	16 (66.7%)
Colonoscopy	6	0	0	0	6 (25%)
Laparoscopy	2	2	1	0	5 (20.8%)
Laparotomy	3	0	2	1	6 (25%)

**Table 4:** Suspected diagnosis of 24 patients with proven abdominal tuberculosis treated at Al-Ain Hospital during the period January 2011 to December 2018

Suspected diagnosis	Number (%)
Abdominal tuberculosis	13 (54.2)
Acute appendicitis	4 (16.7)
Abdominal mass	3 (12.5)
Intestinal obstruction	1 (4.2)
Peritonitis	1 (4.2)
Biliary colic	1 (4.2)
Abdominal wall abscess	1 (4.2)

### Laboratory investigations

C-reactive protein (CRP) was raised in 16 patients (80%), leukocytosis was present in 3 patients (13.63%), and thrombocytosis in 8/22 patients (36.3%) (Table 5). Immunoassay was performed in 14 patients and was diagnostic in 7 of them.

**Table 5:** Blood investigations of 24 patients with abdominal tuberculosis treated at Al-Ain Hospital during the period January 2011 to December 2018

Blood investigations	Value
CRP (normal value < 5 mg/l)	25.1 (2–239)
≥ 5 mg/l	16/20 (80%)
White blood cell count (normal value 4.5–11 × 10^9^/l)	6.65 (3.6–13.7)
> 11 × 10^9^/l	3/22 (13.6%)
ESR (0–20/h)	27 (12–68)
> 20/h	4/8 (50%)
Hemoglobin (normal value 12.1–20 g/dl)	11.7 (7–16.5)
< 12.1 g/dl	12 (50%)
Lymphocytes (normal value 1000–3.500/μl)	1700 (130–2800)
< 1000 μL	3/22 (13.6%)
Platelets (normal value 140–400 × 1000/ml)	338 (156–711)
> 400 × 1000/ml	8/22 (36.3%)

Data are presented as median, range or number (%) as appropriate. Percentages are calculated from available data

### Radiological workup

**Fig. 1:**
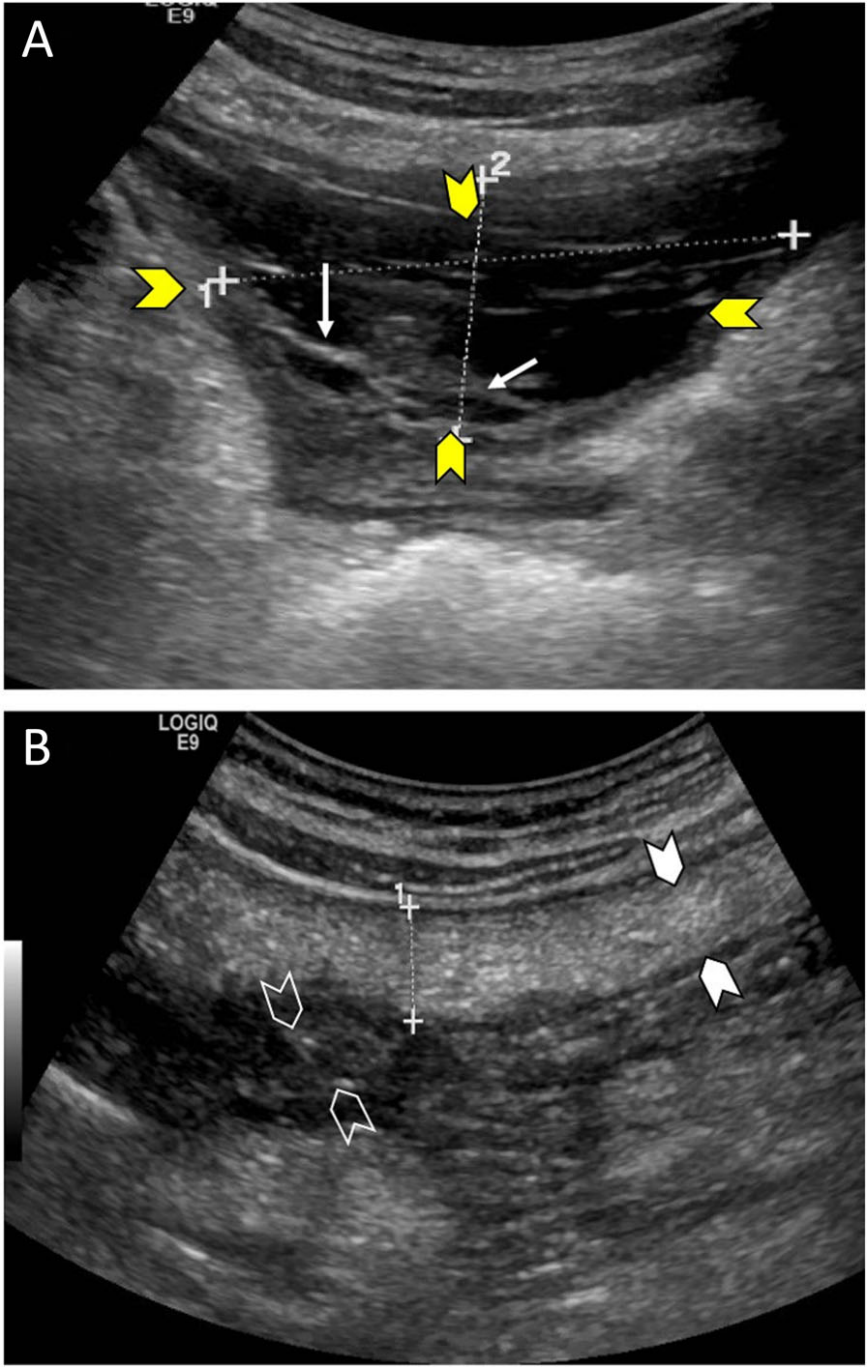
A 29-year-old man presented with shortness of breath, generalized abdominal pain, and fatigue. Abdominal ultrasound scan showed ascites (A, yellow arrow heads) with loculations (A, white arrows) in the pelvis and the left iliac fossa. The peritoneum (B, white arrow heads) and omentum (B, empty arrow heads) were thickened. Peritoneal and pleural fluid cytology were suggestive of an inflammatory tuberculous process. The patient responded to the anti-tuberculous therapy

**Fig. 2:**
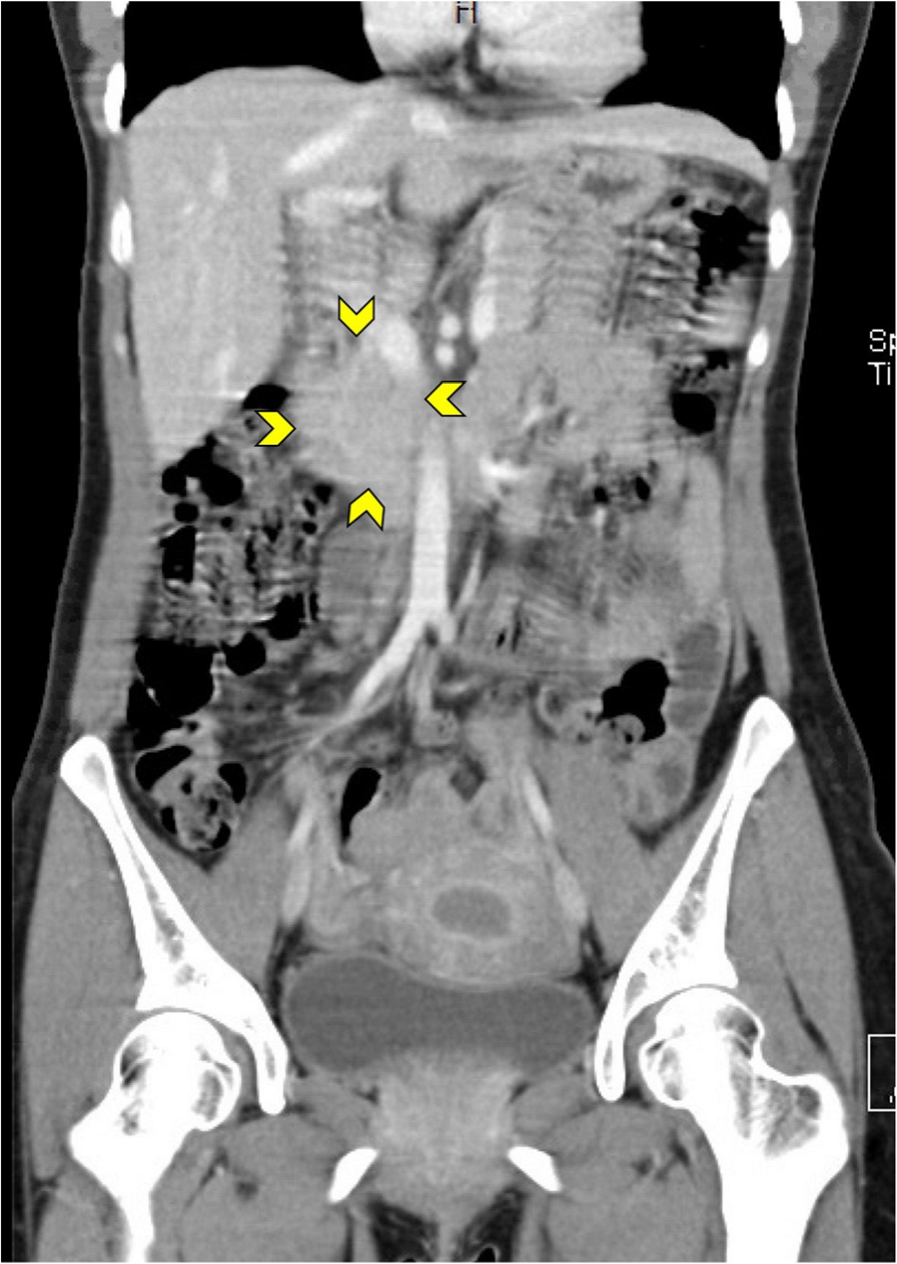
A 29-year-old woman presented with epigastric and lower abdominal pain, fresh vaginal bleeding, and weight loss. Coronal CT scan of the abdomen with intravenous contrast revealed a non-enhanced complex mass lesion measures 4 × 2.5 cm anterior and to the right of the IVC at the level of L3-L4 vertebrae suggesting matted lymph nodes (arrows). Acid Fast Bacilli smear from the mass was positive

**Fig. 3:**
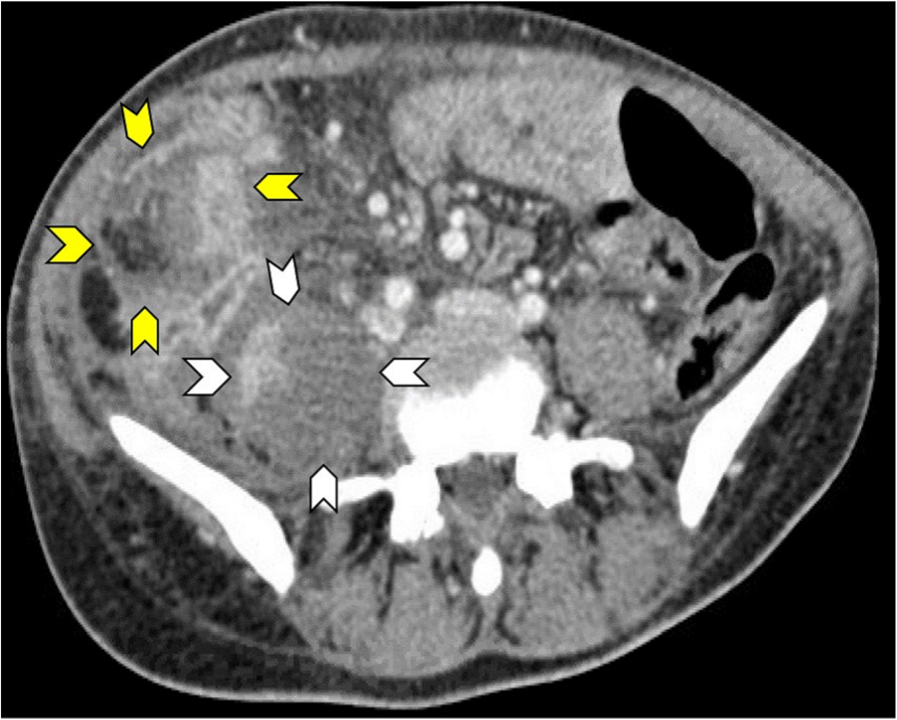
A 31-year-old woman presented with right iliac fossa and right-sided back pain for 1-month duration. She was afebrile, had no cough or night sweats but was anemic. Abdominal CT scan with intravenous contrast showed an inflammatory mass in the right iliac fossa (yellow arrow heads) with sinus tracts communicating to a right iliopsoas abscess (white arrow heads). The patient was from an endemic area of tuberculosis and was treated with anti-tuberculous drugs

Chest X-ray was abnormal in only three patients (12.5%). Two had pleural effusion while the third had left apical pleural scarring. Ultrasound was done in 14 patients and showed abnormal non-specific findings in 11. These findings included free intraperitoneal fluid in 4 patients, intraperitoneal soft tissue masses in three patients, intraperitoneal lymph nodes in three patients, thickened bowel in two patients, thickened omentum in two patients, encysted ascites in one patient (Fig. 1), and abdominal wall mass in one patient. Abdominal CT scan was done in 16 patients. Abdominal tuberculosis was suggested only in three patients: one had an omental cake and adenopathy, the second had a mass suggestive of necrotic tuberculous adenopathy (Fig. 2), and the third had a thickened bowel with suspected pulmonary tuberculosis. Other findings from abdominal CT were non-specific, and included lymphadenopathy in four patients, ileocecal mass in three patients, thickened bowel in three patients, free intraperitoneal fluid in two patients, abdominal cocoon in one patient, thickened omentum in one patient, and iliopsoas abscess in one patient (Fig. 3).

### Colonoscopy

Colonoscopy was performed in 6 patients having ileocecal pathologies. Four had ulcers and two had masses. Biopsies were taken in all 6 patients and were diagnostic in only 2.

### Surgical management

Thirteen patients had surgery. Five had laparoscopy, one had an elective cholecystectomy and biopsy, one had a diagnostic biopsy, three were converted to open surgery of whom two had right open hemicolectomy, while the third had cocoon abdomen which was released. Six patients had laparotomy as the initial procedure. Two needed only a biopsy, two had small bowel resection anastomosis, and two had right hemicolectomy. Two patients had abdominal wall surgery: one had an incision and drainage of an abscess, and one had an excision of a mass. Table 3 shows the summary of the diagnostic workup of the different types of abdominal tuberculosis in our study.

### Final diagnosis

Diagnosis was confirmed by histopathology showing caseating granuloma in 15 patients (62.5%). Two of these patients had acid-fast bacilli on the slides. Immunological assays were diagnostic in 7 patients (29.2%). One patient grew mycobacterium tuberculosis (4%). The workup of one patient was non-conclusive and a therapeutic trial was used to confirm the diagnosis (4%).

### Outcome

Three patients had postoperative complications (12.5%): one patient had wound infection, another had pleural effusion, and a third had a sinus formation. All patients had quadruple anti-tuberculous therapy which included Isoniazid, Rifampin, Ethambutol, and Pyrazinamide for a minimum of 6 months. The patients stayed in the hospital for a median range of 6.5 (0–21) days and were followed up for a period of 6–12 months. One patient needed readmission complaining of vomiting which resolved. None of our patients died.

## Discussion

Our study has shown that the clinical diagnosis of abdominal tuberculosis remains challenging despite advances in medical technology and diagnostic tools. Our patient group was mainly adults in their thirties with equal male to female distribution; abdominal tuberculosis was suspected in only half of our patients: this is similar to other studies [5, 12]. The incidence of abdominal tuberculosis was 0.6 per 100,000 population. The majority of our cases were low-income expatriate workers. Peritonitis, which is a late manifestation of the disease [7–10], occurred in less than 10% of our patients. The speedy development of a well-funded health care system in the United Arab Emirates (UAE) and the diversity of its population make it an ideal setting for studying abdominal tuberculosis. The UAE has a population of 10 million including more than 200 nationalities [13] as shown in Table 1.

Tuberculosis remains a challenging global disease. This paper serves as a reminder for acute care surgeons about the difficulties which may be encountered in its management. Acute care surgeons may find their patients in difficult situations of severe sepsis associated with unresolved bowel obstruction or peritonitis that cannot be explained by the non-specific biochemical and radiological findings. Aiming to help these patients, they may perform emergency laparoscopy or laparotomy only to be surprised by the unfamiliar and unexpected operative findings of abdominal tuberculosis. This can be easily missed as malignancy even by experienced surgeons who are unfamiliar with this disease, which delays the diagnosis of tuberculosis and increases its mortality [10].

Fever was present in less than 40% while leukocytosis was present in less than 15% of our patients. If intra-abdominal pus is found by aspiration or surgery in a healthy-looking patient who does not have fever and leukocytosis, abdominal tuberculosis should be suspected.

The laboratory and radiological workups were not very useful. Anemia and inflammatory markers, although alarming, are not specific [10]. Nevertheless, polymerase chain reaction (PCR) immunoassay tests suggested the diagnosis in seven patients (29.2%). Chest-X-ray was normal in most of our patients. Only 15–25% of abdominal tuberculosis patients have associated pulmonary tuberculosis [14]. Similarly, sonographic findings are nonspecific although they may show ascites, lymphadenopathy, and focal organ lesions [15]. Abdominal CT Scan is more accurate than ultrasound and may indicate the need for further diagnostic measures such as percutaneous aspiration or direct biopsy [15, 16]. It is useful in defining the type and the extent of abdominal tuberculosis [14, 17, 18]. CT scan findings include ascites, peritoneal thickening, lymph node enlargement, bowel wall thickening, and mesenteric fat stranding. These are difficult to differentiate from lymphomas, peritoneal carcinomatosis, and inflammatory bowel diseases [19, 20].

Fine needle aspiration cytology may demonstrate granulomatous cells necessitating the start of early medical therapy which will avoid laparoscopy or laparotomy [12]. In our study, the diagnosis was established histologically in 15 cases (62.5%). The decision to use laparoscopy or laparotomy as a diagnostic method depends on the laparoscopic experience of the surgeon and the type of abdominal tuberculosis. Diagnostic laparoscopy is useful in obtaining tissue samples [21] especially in ascitic peritonitis. Open surgery is indicated in cases of tuberculous abdominal cocoon in order to peel off the fibrous tissue encasing the bowel and hence avoid bowel injury: this procedure was done in one of our patients [22].

Therapeutic diagnosis has a role where there is a high index of suspicion of abdominal tuberculosis and a negative workup [9]. This is especially important when abdominal tuberculosis cannot be differentiated from Crohn’s disease [6]. Treating abdominal tuberculosis with steroids on the assumption that it is Crohn’s disease is very risky and may lead to death [4, 10]. Therapeutic diagnosis was used in around 25% (16–29%) of abdominal tuberculosis in old studies [23–26]. We used this approach only in one patient (4%) which indicates improvements in the diagnostic accuracy. The new immunoassay tests suggested the diagnosis in 7 patients (29.2%). It is possible that 8/24 (33%) would have needed a therapeutic trial if the PCR immunoassay tests had not been available. Accurate PCR results, as reported by advanced tuberculosis research laboratories, cannot be assumed to be reproduced by community-based hospitals. Sampling errors, technical errors, or contamination of the samples in community-based hospitals may give false results [27]. Our results are very encouraging and possibly can be reproduced by other general hospitals. Although PCR is useful in supporting the clinical diagnosis, it cannot differentiate between dead and living mycobacterium because it gives positive results for a long time after the death of the bacteria. Accordingly, PCR should not be used for follow-up [27, 28].

In principle, abdominal tuberculosis should be treated medically, and surgery should be performed only when indicated, which is usually as the last option to reach a diagnosis or to treat complications. Globally, 20–40% of abdominal tuberculosis cases present acutely and need emergency surgery. Indications for surgery include bowel obstruction, perforation, and bleeding [5, 7, 10]. In our study 13 out of 24 patients (54.2%) needed surgery. The median duration of hospital stay in our study was 6.5 days which is less than that reported by others [5]. Despite the presence of comorbidities and associated disease in one fifth of our patients, none of them died, possibly because they were relatively young. The UAE has generally a young working population with very low mortality of severe sepsis at less than 1% [29].

## Limitations

We must acknowledge that our study has certain limitations. *First*, the sample size is relatively small. Nevertheless, it represents all patients treated over 8 years in a major referral hospital for treating tuberculosis with a catchment population of half million inhabitants. *Second*, the study is retrospective in nature which carries the risk of missing data. *Finally*, we could not locate the source of infection which is important for infection control. The majority were expatriate workers suggesting that the infection was carried from overseas although we cannot be sure about that.

## Conclusions

Diagnosis of abdominal tuberculosis remains challenging despite advancements in medical technology and diagnostic tools. The low percentage of the need for diagnostic therapy in our study supports the benefit of PCR assay. Surgery was mainly indicated as the last option to reach the diagnosis or to treat complications.

## Data Availability

Data will be available to the Editor on request.

## Abbreviations

CT: Computed tomography
ESR: Erythrocyte sedimentation rate
PCR: Polymerase chain reaction
UAE: United Arab Emirates
